# Heterogeneity in the prevalence and intensity of bovine trypanosomiasis in the districts of Amuru and Nwoya, Northern Uganda

**DOI:** 10.1186/s12917-015-0567-6

**Published:** 2015-10-08

**Authors:** Harriet Angwech, Jack H. P. Nyeko, Elizabeth A. Opiyo, Joseph Okello-Onen, Robert Opiro, Richard Echodu, Geoffrey M. Malinga, Moses N. Njahira, Robert A. Skilton

**Affiliations:** Department of Biology, Faculty of Science, Gulu University, P.O. Box 166, Gulu, Uganda; Biosciences Eastern and Central Africa (BecA), International Livestock Research Institute (ILRI) - Hub, Old Naivasha Road, P.O. Box 30709- 00100, Nairobi, Kenya; Department of Biology, University of Eastern Finland, P. O. Box 111, 80101 Joensuu, Finland

**Keywords:** Cattle, ITS-PCR, Risk factors, *T. brucei s.l*, *T. congolense*, *T. vivax*, Trypanosomiasis

## Abstract

**Background:**

Livestock trypanosomiasis, transmitted mainly by tsetse flies of the genus *Glossina* is a major constraint to livestock health and productivity in the sub-Saharan Africa. Knowledge of the prevalence and intensity of trypanosomiasis is important in understanding the epidemiology of the disease. The objectives of this study were to (a) assess the prevalence and intensity of trypanosome infections in cattle, and (b) to investigate the reasons for the heterogeneity of the disease in the tsetse infested districts of Amuru and Nwoya, northern Uganda.

**Methods:**

A cross-sectional study was conducted from September, 2011 to January, 2012. Blood samples were collected from 816 cattle following jugular vein puncture, and screened for trypanosomes by HCT and ITS-PCR. A Pearson chi-squared test and logistic regression analyses were performed to determine the association between location, age, sex, and prevalence of trypanosome infections.

**Results:**

Out of the 816 blood samples examined, 178 (22 %) and 338 (41 %) tested positive for trypanosomiasis by HCT and ITS-PCR, respectively. *Trypanosoma vivax* infection accounted for 77 % of infections detected by ITS-PCR, *T. congolense* (16 %), *T. brucei s.l* (4 %) and mixed (*T. vivax*/ *T. congolense/T.brucei*) infections (3 %). The risk of trypanosome infection was significantly associated with cattle age (*χ*^*2*^ *=* 220.4, df = 3, *P* < 0.001). The highest proportions of infected animals were adult males (26.7 %) and the least infected were the less than one year old calves (2.0 %). In addition, the risk of trypanosome infection was significantly associated with sex (*χ*^*2*^ = 16.64, df = 1, *P* < 0.001), and males had a significantly higher prevalence of infections (26.8 %) than females (14.6 %).

**Conclusion:**

Our results indicate that the prevalence and intensity of trypanosome infections are highly heterogeneous being associated with cattle age, location and sex.

## Background

Trypanosomiasis, caused by several species of *Trypanosoma,* is an economically important disease affecting both humans and domestic animals. African trypanosomiasis is transmitted mainly by tsetse flies of the genus *Glossina* known to be distributed widely between latitudes 14°N and 29°S of the equator in 37 Sub-Saharan countries [1]*. Trypanosoma vivax* is also transmitted mechanically by biting flies such as *Tabanus* and *Stomoxys*. The disease excludes livestock and farming systems from about 70 % of the humid and semi-humid zones of sub-Saharan Africa [2]. Schofield and Kabayo [3] estimated the losses to Africa’s Gross Domestic Product (GDP) due to trypanosomiasis infection at about 4.5 billion US dollars annually, including the approximately three million cattle deaths.

Uganda is affected by Human African Trypanosomiasis (HAT) caused by two *Trypanosoma brucei* sub-species: *Trypanosoma brucei gambiense* in the northwest, and *Trypanosoma brucei rhodesiense* in the southeast and northeast of the country [1]. Recent studies have shown the possibility of the two sub-species merging in the country [4]. The epidemiology of HAT is quite complex, with transmission cycles involving interactions between man, tsetse and trypanosomes, and in the case of *T. b. rhodesiense* disease, domestic and wild animals play a significant role as well. In cattle, *Trypanosoma brucei brucei, Trypanosoma congolense* and *Trypanosoma vivax* are species of economic importance.

It has been observed in previous studies that contact between blood feeding insects such as tsetse flies and their respective hosts is highly heterogeneous and non-random [5–7]. As a result, some hosts are bitten more than others, contributing more to the transmission of parasites. The concept of host heterogeneity has been shown to have important implications for disease transmission and to a great extent the design of disease control interventions [8, 9]. Identification of heterogeneities inherent to vector-borne disease systems is a basis for the development of local and adaptive control strategies that efficiently make use of limited resources [10].

Over the years, a number of methods for control of tsetse flies and trypanosomiasis have been developed [11]. These include, treatment of infected animals, aerial spraying, use of animal baits, sterile insect technique, clearing of tsetse infested bushes and slaughtering wild animals. In Uganda, control of trypanosomiasis relies heavily on curative treatment of livestock and the use of tsetse traps. However, there has been significant problem of parasite resistance to trypanocides [12, 13]. Yet the development of new efficacious drugs is considered unprofitable by most pharmaceutical industries considering that this is a disease that affects mainly the rural poor [14]. Thus there is need to develop adaptive control options that are suitable for the resource poor communities in the sub-Saharan Africa. This might be possible if curative treatments target infected animals only, and prophylactic and insecticide treatments target only animals that are challenged most in the herd.

Furthermore, the knowledge of the prevalence and distribution of trypanosome species in a geographical area is essential for understanding the epidemiology of the disease. This information is, however, lacking for the northern Uganda districts of Nwoya and Amuru that have suffered a setback in research due to the prolonged insurgency in the region. The objectives of this study were to (a) assess the prevalence and intensity of bovine trypanosomiasis, and (b) investigate the reason for the heterogeneity of the disease in the tsetse infested districts of Amuru and Nwoya, Uganda.

## Materials and methods

### Study area

**Fig. 1:**
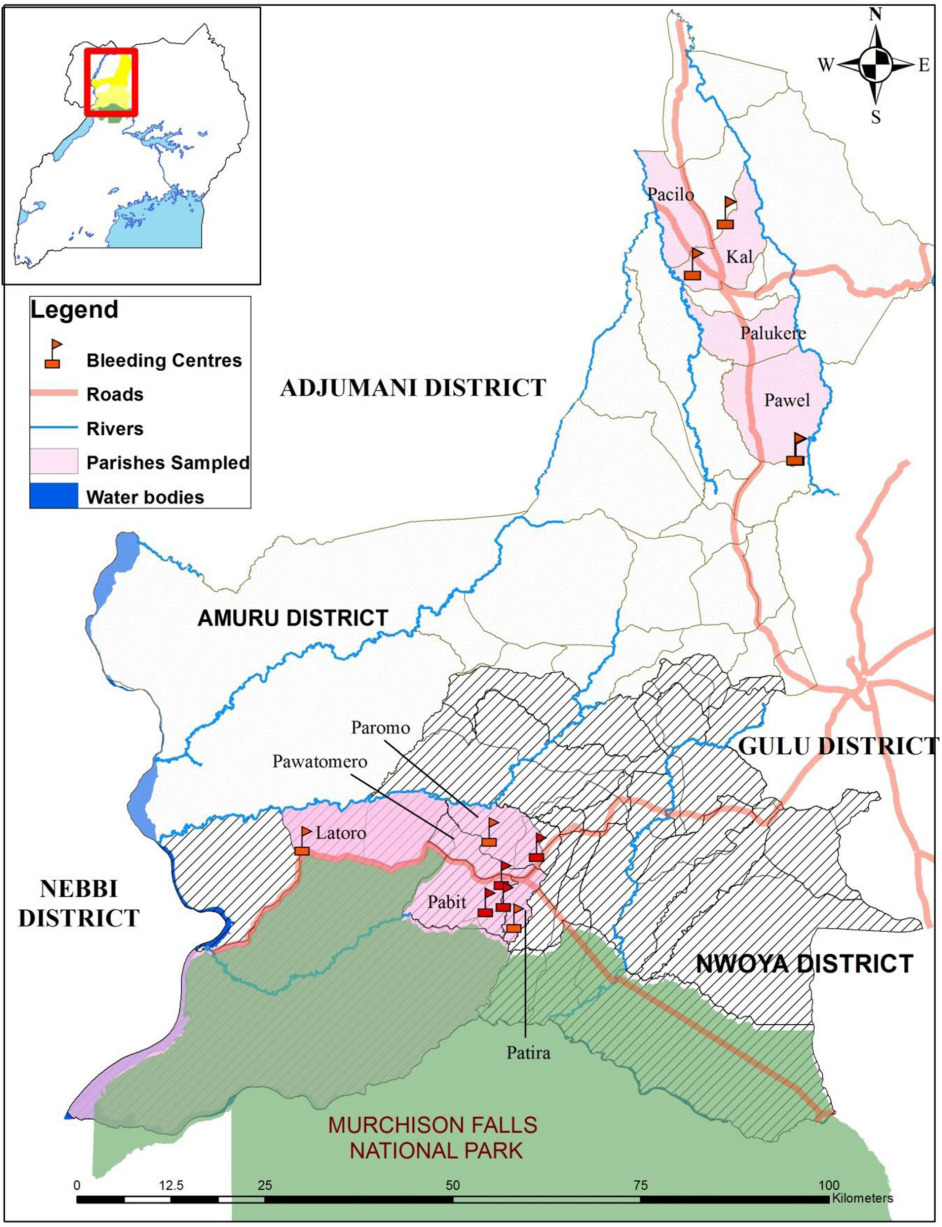
Location of study sites in Amuru and Nwoya districts, northern Uganda (Sampled parishes and bleeding centers). All parishes in Nwoya district included in the study border Murchison Falls National Park (i.e. they are found in the wildlife-livestock interface). Amuru’s parishes on the other hand are located in an area where the density of game animals is low and therefore, livestock constitute the main source of tsetse blood meal

This study was conducted in the districts of Amuru (02° 48' 49"N, 31° 56' 19"E) and Nwoya (02° 38'15"N, 32°00' 53"E) in northern Uganda, that borders the Murchison Falls National Park (Fig. 1). The two districts lie in the animal trypanosomiasis endemic areas and also fall within a *Gambiense* focus with cases of Gambian sleeping sickness reported annually [15]. Subsistence agriculture is the main economic activity of the districts, employing up to 98 % of the population. The arable land constitutes 90 % of the total land area in the districts, and is very fertile (Amuru District Development plan 2011–2016). However, during the last twenty years of prolonged insurgency, there was no form of vector control in place and less than 1 % of the land was utilized for agriculture, and as a result the area became re-invaded by tsetse and other biting flies.

The area has two rainfall periods, July to November and March to May, with a short dry spell in June and a fairly long period of dryness from December to February. The mean annual rainfall is about 1000 to 2000 mm. The average daily minimum and maximum temperatures are 25 °C and 39 °C, respectively [16]. The climate is favourable for agriculture and is conducive for the proliferation of tsetse flies, the vectors for African trypanosomiasis, with *Glossina fuscipes fuscipes* being a major species (99 %) (Angwech *et al*. unpublished observation). Both districts lie within the savannah grassland and are crossed by several rivers and streams. The population density of cattle in the area ranges between 10 and 20 individuals per square kilometer [17].

All parishes in Nwoya district included in the study border Murchison falls national park (i.e they are found in the wildlife-livestock interface), where wild animals used to wander freely in the course of the insurgency when the inhabitants lived in protected IDP camps in trading centers. Cattle are now being reintroduced as part of the national restocking and peace recovery programme of the government of Uganda. The density of livestock, as compared to game animals, is generally low. Therefore, tsetse flies largely depend on game animals for blood meals but also feed on livestock (Angwech *et al*., unpublished observation). Amuru’s parishes on the other hand are located in an area where the density of game animals is low and therefore, livestock constitute the main source of tsetse blood meal.

### Sample size determination

The expected sample size for the number of cattle was determined using the formula provided by Kish [18] assuming a confidence interval of 95 %, a level of expected prevalence of 50 % and an assumed precision of 5 % given that there was no prior information about the prevalence of trypanosomiasis infection in the area. This approach generated an approximate sample size of 816.

### Study design and sampling

A cross-sectional survey design was used to determine the prevalence and intensity of trypanosome species in cattle from September, 2011 to January, 2012. Two sub-counties of Atiak and Purongo in Amuru and Nwoya, respectively, were randomly selected for the study based on the information provided by the district veterinary departments about tsetse infestation in the area and trypanosome-attributable deaths of cattle. From these two sub-counties, nine parishes (Patira, Pawatomero, Latoro, Paromo, Pabit, Kal, Palukere, Pawel and Pacilo) were randomly selected for the study and bleeding centers were established in every parish. Cattle were categorized in to the following age groups: < 12 months; 12 to 48 months and > 48 months. Calves included in this study were only those herded together with other herds under communal grazing system. The ages of cattle were determined based on dentition [19] and information provided by the farmers. All cattle presented for bleeding at the centers were included in the study in order to realize the sample size. In total 816 blood samples (460 males, 356 females) were collected from 19 herds of cattle distributed in the nine parishes of Nwoya and Amuru districts (Patira = 81; Pawatomero = 86; Latoro = 87; Paromo = 103; Pabit = 90; Kal = 87; Palukere = 70, Pawel = 127 and Pacilo = 85).

### Ethical consideration

This research was approved by the Gulu University Institutional Review Board and by the Uganda National Council for Science and Technology. Farmers accepted to participate in the study by signing the consent forms that were provided to them before the animals were bled. Bleeding was done by trained field veterinarians.

### Blood sample collection

The animals used in this study were mainly local Boran short-horn zebu cattle under traditional communal grazing system. For each animal, whole blood sample was drawn into heparinised vacutainer tubes from the jugular vein. Four blood spots (each 120 μl) were made on a single Whatman® Classic FTA Card (Whatman Bioscience, Cambridge, UK) according to the manufacturer’s instructions. The spots were air-dried at ambient room temperature, and the cards stored moisture-free in silica gel awaiting DNA extraction.

### Parasitological tests

Screening of all blood samples for trypanosomes was carried out using haematocrit centrifugation technique (HCT) [20], complemented with direct smears. Parasitological tests for all samples were carried out in the field immediately following blood collection. To perform HCT, three capillary tubes were filled by capillary attraction up to the ¾ mark with uncoagulated blood. Each capillary tube was then sealed at one end using plasticin, centrifuged at 10,000 rpm for 5 min to obtain the buffy coat, and examined for the presence of trypanosomes under a compound microscope (Olympus, magnification × 40). For direct smears, the capillary tubes were cut with a diamond-tipped pencil, and the buffy coat was examined microscopically to detect the presence of motile trypanosomes [21]. This was done for all HCT negative samples. All parasitologically positive animals were given a single curative dose of diminazene aceturate (Berenil®, 3.5 mg kg^−1^ body weight). Parasitaemia scores resulting from trypanosome infections in this study were categorized for intensity using a parasitaemia score from 1 (lowest intensity) to 6 (highest intensity), adopted from ILCA [22]. Scores were assigned as follows: 1[below 10]; 2[10–20]; 3[21–30]; 4[31–40]; 5[41–50] and 6 for more than 50 parasites observed per 50 random fields of view. These values were then organized into classes to ease the analysis and interpretation of results.

### DNA extraction from FTA cards

Using a Harris 3-mm micro-punch (Whatman Biosciences Ltd.), five sample discs were cut randomly from a dried blood spot and placed into a 1.5 ml Eppendorf tube to reduce the chance of missing out trypanosome DNA. To prevent contamination between samples, punches were cleaned after every sampling using a 5 % sodium hypochlorite and 70 % ethanol. The punch was then used to cut a clean filter paper before using it on the next sample. DNA extraction was done using the Invitrogen™ PureLink Genomic DNA kit for purification of genomic DNA (Invitrogen Inc.) following the manufacturer’s instructions. DNA quality and quantity were checked using nanoDrop spectrophotometry and gel electrophoresis. The purified DNA sample was used immediately for PCR analyses or stored at −20 °C until use.

### PCR amplification of DNA

All parasitologically positive and negative extracted DNA samples were subjected to Internal Transcribed Spacers (ITS)1 Polymerase Chain Reaction (PCR) amplification using ITS1 CF (5’CCGGAAGTTCACCGATATTG-3’) and ITS1 BR (5’TTGCTGCGTTCTTCAACGAA-3’) as the forward and reverse primers, respectively. These primers amplify ITS1 region of rDNA genes which are known to vary in size within trypanosome species, and therefore differentiate trypanosome species by their ITS1 sizes [23–25]. All primers were supplied by Bioneer Corporation. The PCR amplifications were performed in a total reaction volume of 20 μL containing 10 μM of each primer, the Bioneer *AccuPower®* PCR premix (Bioneer Corporation), and 2 μL of each DNA template. All PCR amplifications were performed with a thermal cycler (GeneAmp 9700 PCR system, Applied Biosystems). PCR cycling for ITS1 CF and BR primers involved an initial denaturation at 94 °C for 5 min, followed by 35 cycles of 94 °C each for 40 s, 58 °C for 40 s, followed by 72 °C for 90 s, and final extension at 72 °C for 5 min [25]. To ensure that results were not biased by false positives during repeated PCRs, negative controls in which DNA templates were replaced with sterile water as well as positive control DNAs (of each trypanosome species) were included in all PCR reactions. A 4.0 μL of each amplified PCR product was electrophoresed on a 1.5 % agarose gel in 0.5X TBE. A 1 Kb plus DNA size ladder (Fermentas) was included on each gel, stained in Gel Red dye, run constantly at 100 V for 45 min and visualized under ultraviolet trans-illumination. To confirm the results, the selected PCR products for positive samples were column purified using the GeneJet™ PCR purification kit (Fermentas Life Sciences) following the manufacturer’s instructions. Sequencing was done at the BecA-ILRI Hub Sequencing, Genotyping, OligoSynthesis and Proteomics (SEGoLIP) department using both the forward and reverse amplification primers. Sequences were edited in CLC Main Workbench 6.6.5 software (CLC Bio) and blasted to determine their identity. This was done particularly because there were PCR products that produced bands which did not correspond to any known pathogenic trypanosome species band size.

### Statistical analysis

A Pearson chi-squared test and logistic regression analyses were used to determine the association between location, age, sex, and prevalence of trypanosome infections. The threshold for significance was 5 %. All statistical tests were performed using SPSS software (version 16.0.1, SPSS Inc., Chicago, IL, USA).

## Results

Out of the 816 blood samples screened for trypanosome infections, 178 (22 %) and 338 (41 %) animals tested positive by parasitological diagnostic tools (HCT and Direct smears) and ITS PCR, respectively. A total of 77 % of the infections detected by ITS PCR was caused by *T. vivax*, *T. congolense* (16 %), *T. brucei s.l* (4 %) and mixed (*T. vivax*/ *T. congolense/T.brucei*) infections (3 %). All the three *T. congolense* sub-types (*T. congolense ‘*forest’*, T. congolense ‘*kilifi’ and *T. congolense ‘*savanah’) were found infecting cattle in the study area.

### Prevalence of trypanosome infections by location

**Fig. 2:**
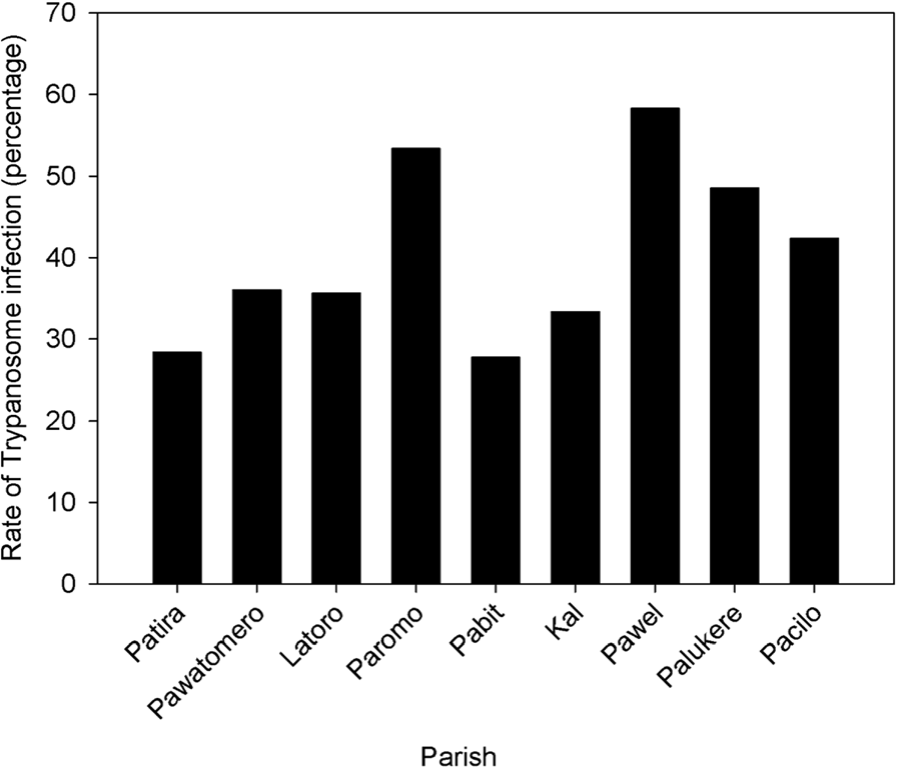
The prevalence of bovine trypanosomiasis in the nine parishes of Amuru and Nwoya districts, northern Uganda

There was a significant association between district location and the prevalence of trypanosome infections (*χ*^*2*^ = 8.28, df = 1, *P* = 0.004). The risk of trypanosome infections was 2.5 times higher in Amuru than in Nwoya district. Furthermore, the prevalence of infections was significantly associated with the parish location (*χ*^*2*^ = 39.59, df = 8, *P* < 0.001). The highest prevalence of trypanosome infections was in Pawel parish in Amuru district and the lowest was in Patira parish, Nwoya district (Fig. 2). The risk of trypanosome infections was 1.6 times higher in Pawel than in Patira parish (OR 2.60; 95 % CI: (1.36–4.98); *P* = 0.004).

### The association between cattle age and trypanosomiasis

There was a significant association between age of cattle and the prevalence of trypanosome infections (*χ*^*2*^ = 73.08, df = 2, *P* < 0.001). The risk of trypanosome infections was nine times higher in males above 48 months of age (OR 10.0; 95% CI: (5.6–17.9); *P* < 0.001) and almost six times higher in cows above 48 months of age (OR 6.1; 95 % CI: (3.8–9.1); *P* < 0.001) than in calves (Table 1). Furthermore, the parasitaemia scores resulting from trypanosome infections were lower in calves than in adults. Table 2 indicates that, in general, 67 % of all detected parasitaemias in calves were scored 1 or 2, i.e. less than 20 parasites observed per 50 random fields of view.

**Table 1:** The prevalence and relative risks of trypanosome infections in cattle of different age categories and sex

Age / sex	*N*	*n*	*n* (%)	Odds ratio (95 % CI)	*P-value*
		HCT	PCR		
Calves (<12 months)	133	09	09 (7)	1.0	reference
Young females (12–48 months)	136	23	48 (35)	5.1 (2.1–9.8)	< 0.001
Young males (12–48 months)	136	27	49 (36)	5.2 (2.4–10.2)	< 0.001
Cows (> 48 months)	168	48	71 (42)	6.1 (3.8–9.1)	0.001
Bulls and oxen (> 48 months)	243	71	161 (66)	10.0 (5.6–17.9)	< 0.001
Total	816	178 (22)^IR-HCT^	338 (41)^IR-PCR^		

*N* number of animals examined, *n* number of positive animals, *(%)* percentage of animals infected, ^*IR-HCT*^ the overall infection rate by HCT, ^*IR-PCR*^ the overall infection rate by PCR. Calves (< 12 months) were used as a reference category

**Table 2:** Relative frequency (%) of parasitaemia scores for trypanosome infections in cattle of different age categories and sex in Amuru and Nwoya districts, northern Uganda

Age / Sex	*N*	Parasitaemia score classes (%)		
		1–2	3–4	5–6
Calves (< 12 months)	09	67	22	11
Young females (12–48 months)	23	43	35	22
Young males (12–48 months)	27	52	26	22
Cows (> 48 months)	48	54	25	21
Bulls and oxen (> 48 months)	71	27	17	56

*N* number of animals infected

### The association between sex and trypanosome infections

There was a significant association between sex of cattle and trypanosome infections (*χ*^*2*^ = 16.64, df = 1, *P* < 0.001). Males were associated with a higher risk of trypanosomiasis (26.7 %) than females (14.6 %; OR 0.49; 95 % CI: 0.36–0.68; *P* = 0.001).

## Discussion

In the present study, we found a significant association between age of cattle and the prevalence of trypanosome infections. A higher prevalence and intensity of trypanosome infections was found in adult cattle (males and females whose ages were greater than 48 months) than in calves less than twelve months (Table 1). Similar results have been found in several previous studies [6, 26–29]. This could be attributed to differences in the period of exposure to vectors between the cattle age groups [28]. In traditional grazing systems, suckling calves are restricted or grazed at homesteads and not allowed to graze with adults until weaned off [27]. This practice minimizes their chances of contact with the disease vectors when compared to adults. Additionally, calves are known to be protected by the effect of maternal immunity, which helps to keep parasitaemia low to almost undetectable levels [30]. This is further confirmed by the findings of this study, where up to 67 % of infected calves had lowest parasitaemia scores (Table 2). The association between the prevalence of trypanosome infections and age of cattle might also be explained by differences in the attractiveness of animals of different ages to tsetse flies [7]. Studies on host preference have shown that tsetse flies are attracted most to odours of larger or older animals and least to calves [31].

The findings from this study have also indicated a significant association between sex and prevalence of trypanosome infections. Males had a significantly higher prevalence and risk of trypanosome infection than female cattle. The finding is consistent with Magona *et al.* [32] who showed that male cattle had a significantly higher prevalence of trypanosomes than females. In contrast, other studies [29, 32, 33] found no significant differences in the prevalence of trypanosome infections between males and females. Magona *et al*. [32] attributed this variation to the functional status of the animal such as being draught ox due to heavy stress from their use in ploughing. This stress condition might contribute to increased susceptibility of male animals to trypanosome infections.

*Trypanosoma vivax* was found to predominate in the study area and contributed up to 77 % of infections in cattle. This is in agreement with the findings of Magona *et al.* [34] who also indicated *T. vivax* as the most predominant trypanosome species in the *Glossina fuscipes fuscipes* infested areas. This could be explained by the vectorial capacity of *G. fuscipes fuscipes* [35] that accounts for over 90 % of tsetse species infesting the study area (Angwech *et al.* unpublished observation). Earlier studies have reported differences in vector competence and ability to transmit trypanosomes particularly between *G. f. fuscipes* and *G. pallidipes. Glossina f. fuscipes* reported to be a poorer transmitter of *T. congolense* than *G. pallidipes* was shown to have a higher transmission index for *T. vivax* compared to *G. pallidipes* [35]. In addition, *T. vivax* is known to have the shortest life cycle among all the trypanosome species recorded so far [36]. The predominance of *T. vivax* in the study area is consistent with the findings of several previous studies in Uganda [29, 37, 38]. The two districts are currently under the cattle restocking progamme of the government of Uganda and support of the non-governmental organizations working in the region. The fact that all the three types of *T. congolense* were detected in cattle is not surprising. This could be due to the importation of infected animals from other geographical areas through the restocking programme [15].

ITS-PCR detected a higher prevalence of trypanosome infections (41 %) compared to parasitological tests; Haematocrit centrifuge technique (22 %). Haematocrit centrifuge technique is limited by having a low resolution and sensitivity that require a high threshold of detection of trypanosomes. PCR on the other hand, has a high sensitivity and specificity that considerably reduces the threshold levels of detection of infection to as low as one trypanosome per milliliter of blood [39, 40]. Besides, both districts are found in a trypanosomiasis endemic focus where most of the infections present in a chronic form characterized by low parasitaemia in which case PCR would be two to three times more sensitive. This further confirms the importance of PCR in epidemiological studies over parasitological methods.

## Conclusion

In conclusion, the results of the present study indicate that the prevalence and intensity of trypanosomiasis infections are highly heterogeneous being associated with cattle age, sex and location.

### Limitations of the study

The major limitations of this study were: firstly, it was not possible to document the enrolment of cattle or where they were brought from into the study area after the insurgency/conflicts. This was partly because some farmers who received the animals as donations from the support NGOs and the government did not know exactly where they came from.

Secondly, only the parasitologically positive animals were treated with a curative dose of diminazene aceturate due to the limited number of personnel involved in the field data collection and also due to the delay in PCR identification of the parasites.

Thirdly, even though PCR yielded bands which never corresponded to any known pathogenic trypanosome species band size were identified as trypanosomes, their profiles were different from the wild profile, and thus we recommend further investigation using more sensitive methods such as PCR- restriction fragment length polymorphism which are able to detect intra-species as well as interspecies variation to clarify this situation.

## Abbreviations

ITS-PCR: Internal Transcribed Spacer - Polymerase Chain Reaction
HAT: Human African Trypanosomiasis
HCT: Haematocrit Centrifugation Technique
BecA-ILRI: Biosciences Eastern and Central Africa – International Livestock Research Institute
SEGoLIP: Sequencing, Genotyping, Oligosynthesis and Proteomics
MSI: Millennium Science Initiative
TBE buffer: Tris-Borate-EDTA buffer
DNA: Deoxyribonucleic Acid
